# How to Remove Wax from the Pins of Teeth Prior to Packing with Rubber

**Published:** 1869-12

**Authors:** B. M. Wilkerson


### ARTICLE IV.

### *How to Remove Wax from the Pins of Teeth Prior to Packing with Rubber.*

By B. M. Wilkerson, D.D.S.

After separating the flask, place the half which has the teeth in it in a basin ; then pour *boiling* water (a quart will be sufficient) in a large stream on the pins at a distance of three or four feet. Use the precaution to have the flask warm to prevent cracking the teeth. If properly done it will remove all the wax a *great* deal better than boiling, and *much* sooner.

